# The essential role of long non-coding RNA GAS5 in glioma: interaction with microRNAs, chemosensitivity and potential as a biomarker

**DOI:** 10.7150/jca.49203

**Published:** 2021-01-01

**Authors:** Xuewen Tan, Haifeng Jiang, Yilong Fang, Dafei Han, Yawei Guo, Xinming Wang, Xun Gong, Wenming Hong, Jiajie Tu, Wei Wei

**Affiliations:** 1 Institute of Clinical Pharmacology, Anhui Medical University, Key Laboratory of Anti-Inflammatory and Immune Medicine, Ministry of Education, Anhui Collaborative Innovation Center of Anti-Inflammatory and Immune Medicine, Hefei, China.; 2 The First Affiliated Hospital of Anhui Medical University.

**Keywords:** GAS5, miRNA, glioma, chemosensitivity

## Abstract

Glioma is a malignant brain tumor with a generally poor prognosis. Dysregulation of a long non-coding RNA, GAS5, has been detected in numerous cancers, including glioma. Previous studies have suggested that GAS5 plays a significant functional role in glioma, affecting proliferation, metastasis, invasion, and apoptosis. In this review, we describe the roles and mechanisms of GAS5 in glioma. GAS5 may be a biomarker for diagnosis and prognosis, and even a potential target for glioma treatment, and therefore warrants further investigation.

## Introduction

Glioma is a malignant brain tumor which is highly migratory and invasive, and has high mortality 1. Glioma patients undergoing conventional treatment, including surgery, radiotherapy, and chemotherapy, have poor prognosis 2. It is therefore important to develop new approaches to treatment. Studies into the pathogenesis of glioma may contribute to the discovery of new therapeutic strategies. Recently, several reports have focused on the pathogenesis of glioma, especially with respect to epigenetics 3, 4, an approach that provides a novel perspective on glioma pathogenesis and potential treatment.

Long non-coding RNAs (lncRNAs) are involved in the pathogenesis of a range of diseases, including cancers. lncRNAs are >200 nucleotides long, and have low protein-coding potential 5. lncRNAs participate in the pathogenesis and behavior of cancer by interacting with DNA, miRNAs, and proteins 6. Depending on the nature of the cancer, lncRNAs can act as either tumor suppressor lncRNAs, including lncRNA taurine up-regulated gene 1 (TUG1) 7 and lncRNA-maternally expressed gene 3 (MEG3) 8, or onco-lncRNAs, such as lncRNA HOX transcript antisense intergenic RNA (HOTAIR) 9, urothelial carcinoma associated 1 (UCA1) 10 and lncRNA nuclear paraspeckle assembly transcript 1 (NEAT1) 11. These lncRNAs may be potential targets for the treatment of tumors. Recently, several studies have demonstrated the role of lncRNAs in the pathogenesis of glioma 12-14; however, the underlying molecular mechanisms remain unclear.

The lncRNA known as growth arrest-specific 5 (GAS5) is encoded by the *GAS5* on chromosome 1q12.1 15, 16. Aberrant GAS5 expression has been detected in several cancers, including breast cancer, 17 and gastric cancer 18. Recently, the role of GAS5 has also been reported in the pathology of glioma. Here we summarize recent studies that report the role of GAS5 in glioma, and describe the known interactions between GAS5 and miRNAs, and other molecular pathways. Evidence for a correlation between GAS5 and chemosensitivity, and for potential roles for GAS5 in the diagnosis and treatment of glioma, is also presented.

## Interactions between GAS5 and miRNAs in glioma

GAS5 is involved in the development of numerous diseases, especially cancer. GAS5 acts as a tumor suppressor in most neoplastic diseases. Several studies had found that GAS5 expression levels were lower in glioma cells than in normal controls 19. GAS5 had been found to be downregulated in different stages of glioma, and was at its lowest in advanced glioma patients 20. However, understanding the mechanism of action of GAS5 in the pathogenesis of glioma requires further research.

Bioinformatics analysis predicts the presence of several miRNA binding sites on GAS5, indicating that GAS5 may negatively regulate the occurrence and development of cancers by targeting miRNA. Luciferase reporter assays had shown that miR-222 overexpression significantly reduced luciferase activity in the luciferase reporter plasmid, suggesting that miR-222 was one of the targets of GAS5. The tumor suppressors Bcl-2-modifying factor (Bmf) and Plexin C1 (PLXN C1), both of which were expressed at low levels in glioma, had been identified as targets of miR-222. Overexpression of GAS5 or down-regulation of miR-222 regulated the expression of the apoptosis regulator Bcl-2 family, including down-regulated Bcl-2 and up-regulated Bax via up-regulating Bmf. Overexpression of GAS5 or down-regulation of miR-222 also inactivated the actin-binding protein cofilin by increasing the amount of PLXN C1 20. Scott *et al.* 21 also demonstrated that PLXN C1 stimulated the synthesis of Lim kinase II (LIMKⅡ) and thus promoted the phosphorylation of cofilin. In another study, miR222/221 was found to be highly expressed in high-grade glioma, and levels of protein tyrosine phosphatase μ (PTP μ) were low. miR-222/221 overexpression negatively regulated PTPμ and hence enhanced the movement of glioma cells^22^. In addition, a colorectal cancer study reported that miR-222/221 could activated NF-κB and signal transducer and activator of transcription-3 (STAT3). Moreover, miR-222/221 bound to the coding area of RELA mRNA, and enhanced RELA mRNA stability. miR-222/221 also inhibited PDLIM2 (PDZ and LIM domain 2), therefore inhibiting RELA and STAT3 protein ubiquitination and degradation 23.

Zhao *et al.* 24 found that miR-196a-5p expression was increased in glioma stem cells and acted as an oncogene. GAS5, which bound to miR-196a-5p, exhibited an effect opposite to that of miR-196a-5p. Silencing miR-196a-5p positively regulated the expression of forkhead box protein O1 (FOXO1). FOXO1 increased the expression of phosphotyrosine interaction domain containing 1 (PID1), which inhibited tumorigenesis and growth. FOXO1 also enhanced the expression of migration and invasion inhibitory protein (MIIP), which decreased the migration and invasion of glioma cells. A positive feedback loop between FOXO1 and GAS5 had also been demonstrated. MIIP bound to histone deacetylase 6 (HDAC6) and negatively regulated the stability of its protein, inhibiting cell movement 25. MIIP has also been reported to play a role in other cancers. For example, MIIP promoted epidermal growth factor receptor (EGFR) protein degradation via the proteasome and lysosome pathways. MIIP facilitated the binding of EGFR protein and BIP, an endoplasmic reticulum chaperone, thereby activating the proteasome degradation pathway, leading to the degradation of newly synthesized EGFR. MIIP also negatively regulated the expression of HDAC6, resulting in the degradation of mature EGFR protein via the lysosome degradation pathway. Finally, two pathways for EGFR protein degradation modulated the stability of EGFR and inhibited the EGFR/Ras/MER/ERK signal pathway, leading to reduced proliferation of lung cancer cells 26.

Ding *et al.* 27 discovered that GAS5 could repress miR-10b expression to further suppress tumor exacerbation. GAS5-downregulated miR-10b reduced the expression of Sirtuin 1, thereby hindering the phosphorylation of phosphoinositide 3-kinase (PI3K), protein kinase B (AKT), mitogen-activated protein kinase kinases (MEK), and extracellular signal-regulated kinase (ERK), and increasing the expression of phosphatase and tensin homolog (PTEN). Inhibited miR-10b also enhanced Bax expression, while decreasing the expression of Bcl-2.

A GAS5 mimic decreased the levels of miR-18a-5p, and a miR-18a-5p mimic reduced GAS5 expression in glioma. miR-18a-5p directly bound to exon-2 of GAS5 and inhibited the progression of glioma to a certain extent 28. miR-34a had also been identified as a target of GAS5 in malignant glioma and other cancer types, such as renal cell carcinoma and hepatocellular cell carcinoma. GAS5 also participated in the pathological processes including survival, the cell cycle, metastasis and invasion of tumor cells, by interacting with miR-34a 29.

**Figure 1:**
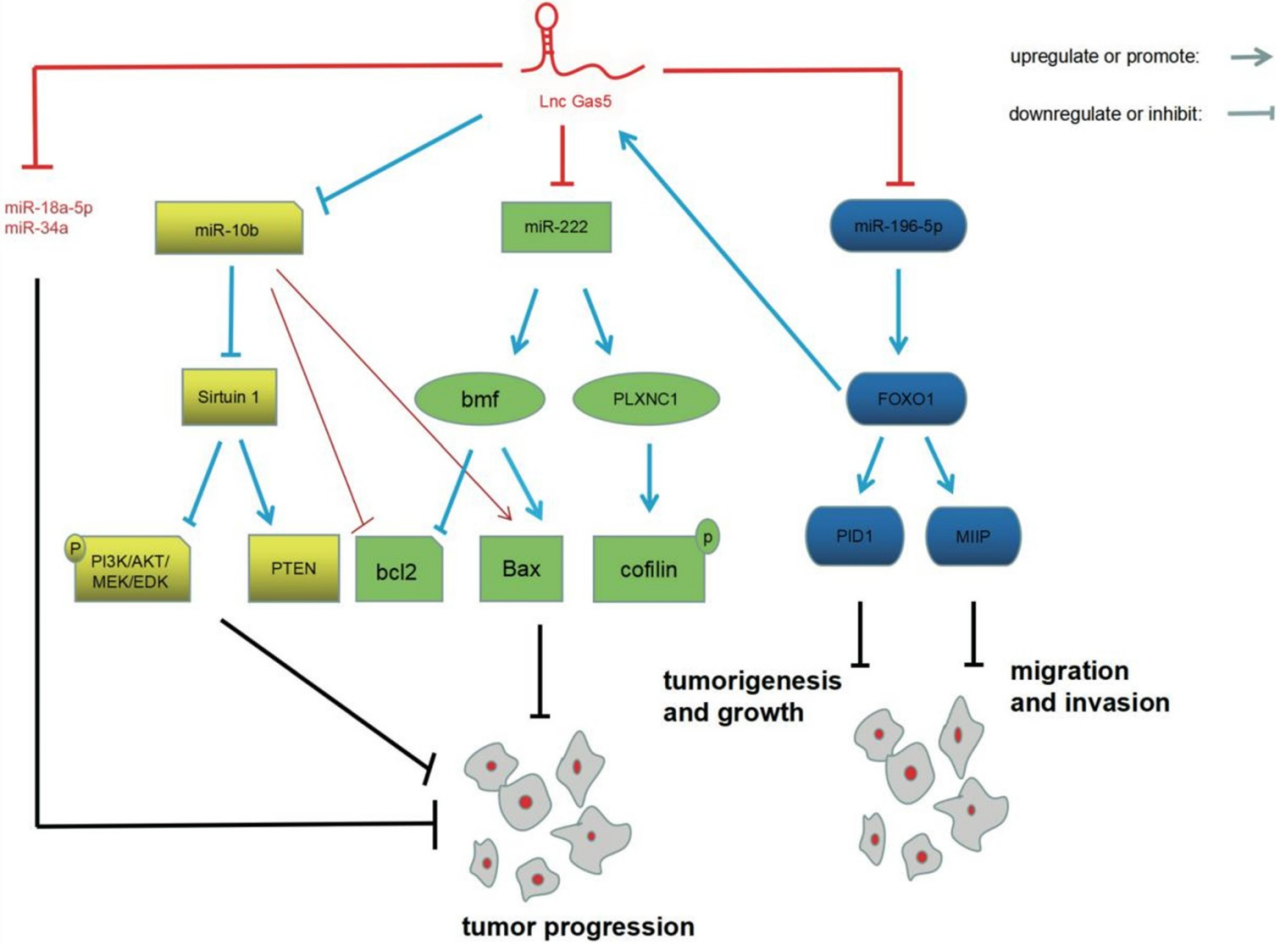
Interactions of GAS5 and miRNAs in glioma pathogenesis. GAS5 inhibits the expression of miRNAs, including miR-222, miR-196-5P, miR-10b, miR-18a-5p, and miR-34a. GAS5-targeted miR-222, GAS5-targeted miR-196-5p, and GAS5-targeted miR-10b are involved in the pathogenesis of glioma through bmf/bcl2/Bax/PLXN C1/cofilin, FOXO1/PID1/MIIP and sirtuin 1/PI3K/AKT/MEK/ERK/PTEN, respectively.

In summary, GAS5 exerts a tumor suppressive effect in glioma through interactions with miRNAs (**Figure 1**). Not only that, a large number of studies suggested that GAS5 inhibits tumor progression in other cancers, including cervical cancer 30, liver cancer 31 and bladder cancer 32. However, A few studies reported GAS5 exhibits the opposite effect. For example, GAS5-007, which is one of the transcripts of GAS5, promotes cell proliferation and inhibits cell apoptosis of prostate cancer 33. miRNAs, key players in the pathogenesis of glioma, are essential targets of GAS5. The research described above indicates that GAS5-targeted miRNAs are involved in tumorigenesis and the progression of glioma. In addition, GAS5-targeted miRNA has also been reported in other tumors. For example, GAS5/miR-106a-5p/Akt/mTOR (mammalian target of rapamycin) has been reported in gastric cancer 34. A bladder cancer study discovered GAS5/miR21/PTEN pathway 32. GAS5-targeted miRNAs and downstream signaling pathways are extremely complex and require further in-depth research.

## GAS5 and chemosensitivity in glioma

Conventional treatment for glioma includes chemotherapy. However, resistance to chemotherapy often interferes with the effects of treatment. Temozolomide (TMZ), which was the first-line chemotherapy drug for glioma, was subject to both natural resistance and resistance acquired during treatment 35. New adjuvant therapy drugs had shown anti-cancer activity *in vitro* experiments, but had limited efficacy when used in patients with glioma. For example, only 10-20% of patients benefited from EGRF inhibitor treatment 36. Although cisplatin (DDP), doxorubicin (DOX), and vincristine (VCR) were recognized anti-tumor drugs, resistance to DDP, DOX, and VCR in the treatment of glioma had been reported 37-39. Recently, studies have shown that GAS5 is a potential therapeutic target in glioma, and may be related to chemotherapy resistance during treatment.

**Figure 2:**
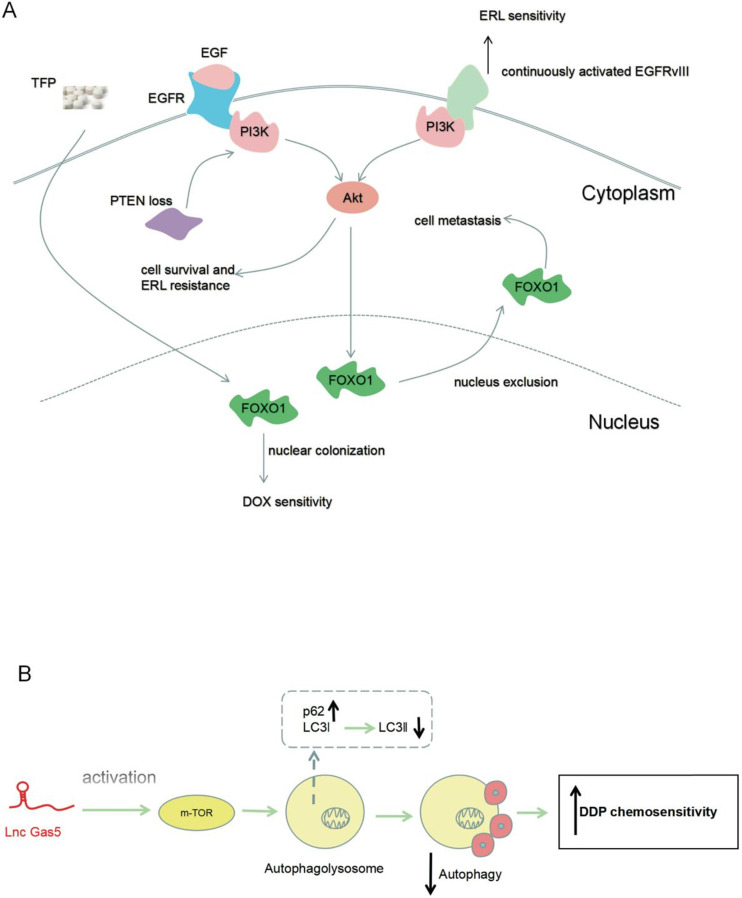
Role of GAS5 in improving chemosensitivity in glioma. (**A**) PTEN loss promotes resistance to ERL chemotherapy, but activated EGFRvIII enhances ERL chemosensitivity. TFP enhances DOX chemosensitivity by FOXO1 chemosensitivity. (**B**) GAS5 inhibits drug-induced autophagy by activating the m-TOR signal pathway, leading to increased sensitivity to chemotherapy. During this period, the expression of autophagy substrate P62 increases and LC3II expression decreases.

Liu *et al*. 40 found that GAS5 was upregulated by the cytotoxic effects of doxorubicin in glioma cell lines (U251 and U87). However, GAS5 levels did not change in the cytotoxic treatment of resveratrol. These results suggested that variation of GAS5 occured during treatment with specific chemotherapy drugs, and was connected with chemosensitivity in glioma. Trifluoperazine (TFP) regulated FOXO1, increasing the amount of FOXO1 in the nucleus, and thus increasing the cytotoxicity of doxorubicin 41. It had also been reported that a combination of EGFR and EGF activated the PI3K/Akt signaling pathway, induced FOXO1 nuclear exclusion, and promoted the metastasis of glioblastoma 42. These observations indicate that FOXO1 nuclear leakage may be involved in chemoresistance in glioma (**Figure 2A**).

Erlotinib (ERL), an EGFR tyrosine kinase inhibitor, is a second-line drug for glioma treatment. Endogenous expression of GAS5 increased during erlotinib treatment in glioma, implying that GAS5 might be relevant to the resistance of glioma cells to erlotinib 43. ERL inactivated EGFR and EGFR deletion mutant variant III (EGFRvIII), thereby inhibiting the phosphatidylinositol 3-kinase/Akt (PI3K/Akt) signal pathway. Inactivated Akt signal inhibited the survival and proliferation of tumor cells. However, an absence of PTEN, an inhibitor of the PI3K signaling pathway, reversed the inactivation of Akt caused by erlotinib. Finally, the tumor cells became less sensitive to erlotinib 44, 45. GAS5-targeting of miR-10b also could lead to increased PTEN expression 27 (**Figure 2A**).

Huo *et al.* 46 demonstrated that glioma cells with enhanced GAS5 expression had higher sensitively to cisplatin, however, silencing GAS5 in glioma cells produced adverse effects. GAS5 overexpression in U138 cells activated the mTOR signal pathway, thereby hindering the expression of LC3Ⅱ (marker of autophagic flux), and increasing the expression of p62 (autophagy substrate). An active mTOR signal pathway restrained excessive autophagy, which was induced by cisplatin, and thus improved chemosensitivity. In contrast, repressed GAS5 U87 cells exhibited reduced chemosensitivity due to inhibition of the mTOR pathways (**Figure 2B**).

Unsatisfactory treatment of glioma is largely due to drug resistance. Studies indicate that FOXO1, PTEN, Akt, PI3K, and mTOR may be closely involved in chemotherapy resistance in glioma. GAS5 can modulate these targets directly or indirectly. Therefore, GAS5 may be a key factor causing drug resistance in glioma, but its downstream pathways need in-depth study. Several reports have suggested that GAS5 may be a target for treatment of glioma, involved in overcoming drug resistance, and for increasing chemosensitivity in other cancers. Gu *et al.* 47 found that low expression levels of GAS5 were detected in MCF-7R cells, a breast cancer cell line, increasing the resistance to tamoxifen. MCF-7R cells overexpressing GAS5 showed higher sensitivity to tamoxifen, while GAS5 down-regulation decreased the sensitivity of MCF-7R cells to tamoxifen. GAS5 overexpression also produced greater sensitivity to cisplatin in two ovarian cancer cell lines, HEY and SKOV3. Moreover, the overexpression of GAS5 also produced increased chemosensitivity to cisplatin *in vivo* in a tumor formation model in nude mice 48. Taken together, these results indicate that GAS5 could improve the sensitivity of glioma tumor cells to chemotherapy, thereby enhancing the effectiveness of treatment.

## GAS5 as biomarker for glioma

Research had indicated that single nucleotide polymorphisms (SNPs), such as polymorphisms in the genes for kinase-anchored protein 6 (AKAP6) 49, EGFR 50, and HOTAIR 51, were associated with susceptibility to glioma and consequent prognosis. A polymorphism in *GAS5*, known as rs145204276, had been connected with glioma susceptibility; patients who carried a GAS5 rs145204276 del allele had a higher chance of having glioma. The GAS5 rs145204276 polymorphism interfered with the binding capacity of TFAP2A, a transcriptional factor and an established tumor suppressor of glioma 52.

Obtaining an accurate prognosis for glioma is essential due to its high relapse rate. Wang *et al.* 53 found that risk factors in glioma patients included isocitrate dehydrogenase 1 (IDH1) mutations, histological grade, tumor size, histological stage, gender, age, and GAS5 expression. Specifically, GAS5 expression in patients with an IDH1 mutation was significantly higher than in patients with a wild-type IDH1. GAS5 levels could effectively predict the survival rates of low-grade glioma, including grades II and III. Low-grade glioma patients with high GAS5 expression had a higher probability of survival. An association of GAS5 with overall survival was not found in glioblastoma.

Shen *et al.* 54 suggested that GAS5 overexpression might be related to decrease in death, recurrence, and progression, but high levels of HOTAIR produced the opposite result in glioblastoma. These results indicated that investigating the expression of GAS5 and HOTAIR in combination could produce more accurate predictions about the survival, recurrence, and progression of glioblastoma. Studies had also shown that GAS5 rs145204276 and HOTAIR rs4759314 polymorphisms affected the expression of GAS5 and HOTAIR, affecting the survival rates of prostate cancer. GAS5-targeted miR-1284, and HOTAIR-targeted miR-22 acted on high mobility group box 1 (HMGB1) 55.

The C/D box small nucleolar RNA U76 (SNORD76), which was derived from intron 3 of the GAS5 DNA sequence, was expressed at low levels in primary glioma tissues and glioma cells. Chen *et al.* 56 found that SNORD76 induced cell cycle arrest in S phase in glioma cells by affecting cell cycle-related proteins. Rb and pRb were upregulated, and cyclin A1, cyclin B2, and p107 were downregulated, leading to reduction in the malignancy of glioma. The small nucleolar RNU44 (SNORD44) was one of the products of the GAS5 gene. Similar to GAS5, SNORD44 was expressed at low levels in glioma cells. SNORD44 accelerated apoptosis and suppressed tumor deterioration by activating the caspase-dependent apoptotic pathway 57.

**Figure 3:**
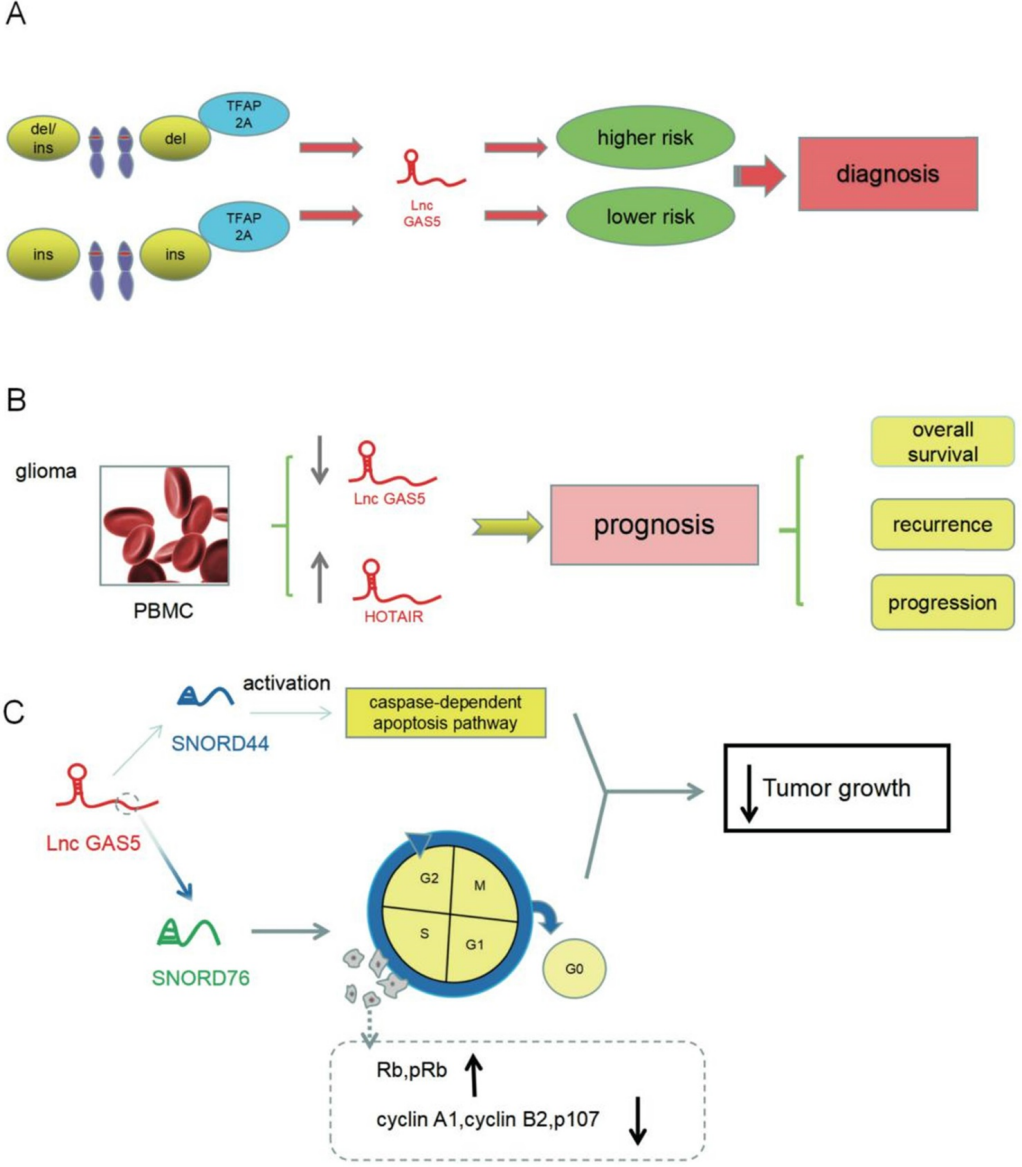
Predicted role for GAS5 in the diagnosis and prognosis of glioma. (**A**) Polymorphism in the GAS5 gene can lead to a preliminary diagnosis of susceptibility to glioma by TFAP2A. (**B**) Expression of GAS5 contributes to an understanding of the prognosis of glioma. (**C**) GAS5 products SNORD76 and SNORD44, which may be targets for treatment of glioma, inhibit tumor growth by affecting cell cycle-related proteins and the caspase-dependent apoptosis pathway, respectively.

GAS5, which affects tumor susceptibility to glioma and overall survival, may be a target for diagnosis and prognosis in glioma. GAS5 dysregulation in other cancers 32, 58 and non-tumor diseases 59, 60 had also been reported. GAS5 is not a specific predictor of glioma, but can be used to predict the prognosis for glioma after excluding other diseases, or by combining it with other predictors. GAS5 as a target for diagnosis and prognosis needs further research (**Figure 3**).

## Conclusions and perspective

Glioma is the most malignant tumor occurring in the brain, and has a generally poor prognosis. In order to improve the survival rates of glioma patients after treatment, research into etiology, pathogenesis, and drug resistance mechanisms of glioma is key. In this review, we describe the mechanisms by which lncRNA GAS5 is known to be associated with glioma pathogenesis and drug resistance. However, the role of GAS5 in glioma is complex, and there are direct or indirect effects on a range of cancer-related genes. According to the DIANA tools (http://diana.imis.athena-innovation.gr/DianaTools/index.php) and starBase (http://starbase.sysu.edu.cn), 46 kinds of miRNA and GAS5 were found to have complementary base pairing 28. GAS5 also has other targets, including RNAs, DNA, and proteins. GAS5 affected the behavior of glioma by downregulating glutathione-S-transferase M3 (GSTM3), including negatively regulating glioma cell proliferation, metastasis, and invasion, and leading to apoptosis and reactive oxygen species generation 19. GAS5 also inhibited the expression of STAT3, suppressing T helper 17 cell (Th17) differentiation in immune thrombocytopenia (ITP) 61. It is difficult to systematically analyze the regulatory network of GAS5 in glioma, but an understanding of this system is important, and should be a research focus in the future.

We can improve our understanding of GAS5 regulatory pathways in glioma in two ways. We can obtain new information about the regulatory mechanisms of GAS5 in glioma from studying other tumor diseases. The expression of miR-21, which was suppressed by GAS5, reduced SPRY2 expression in ovarian cancer 62. GAS5/miR-21/SPRY2 may act on glioma cells and is of value for further research. There are many anti-tumor drugs based on various targets, while drug resistance often leads to treatment failure. Compared with the traditional strategy of targeting oncogenes, improving the sensitivity of chemotherapy may be more helpful to improve the treatment effect. Moreover, GAS5 enhanced chemotherapy sensitivity of adriamycin in breast cancer 63 and improved the efficacy of cisplatin in non-small cell lung cancer 64. miR-222 suppressed by GAS5 enhanced the expression of PTEN and thus improved the chemosensitivity of breast cancer to tamoxifen 47. GAS5 targeted miR-222 and its downstream signaling, which was involved in the development of glioma 20. Therefore, GAS5/miR-222/PTEN may also be important in chemosensitivity in glioma. FOXO1 had been reported to affect tumor-associated macrophage (TAM) polarization 65. GAS5-targeted miR-196-5p also affected FOXO1 in glioma 24. It is therefore clear that research into the role of GAS5/miR-196-5p/FOXO1 in tumor-associated macrophages in glioma is needed. We can also apply new methods to GAS5 research. Pang *et al* 66 investigated the invasion pathways of glioblastoma, and the key molecules involved in this process, using single-cell RNA assays. Developing technologies such as single-cell RNA sequencing may provide new insights into the underlying mechanisms of glioma, including the role of GAS5. Both established and new technologies should be applied to further research into the mechanisms of action of GAS5 in glioma.
